# Prostate Cancer – Local Treatment after Radiorecurrence: Salvage Cryoablation

**DOI:** 10.1590/S1677-5538.IBJU.2018.03.05

**Published:** 2018

**Authors:** Rodrigo Donalisio da Silva, Fernando J. Kim

**Affiliations:** 1 Division of Urology, Denver Health Medical Center, Denver, CO, USA; 2 Division of Urology, University of Colorado Denver, Denver, CO, USA

**Keywords:** Radiation, Prostatic Neoplasms, Radiotherapy

Radiation therapy (RT) is an effective treatment for localized prostate cancer and approximately 45% of patients diagnosed with localized prostate cancer choose radiotherapy as initial treatment (1). Novel RT technology is evolving, allowing more targeted and higher doses of radiation in the prostate such as three-dimensional conventional RT and intensity-modulated conformal RT.

Currently, PSA is the preferred method to monitor patients after prostate cancer RT treatment, but up to 63% of patients may have elevated PSA within 10 years after RT (2). Ray et al. showed biochemical recurrences (PSA criteria) were 33%, local failure 9%, and distant failure (metastasis) 7% in a multi-institutional study of patients with prostate cancer primarily treated with RT that received 60 Gy or higher. Therefore, it is estimated that annually in the U.S., 45,000 patients will have asymptomatic biochemical recurrence following primary RT (1). For patients with intermediate and high-risk prostate cancer, biochemical recurrence is around 50% within 10 years of follow-up (3).

Patients with biochemical (ASTRO or Phoenix criteria) recurrence after RT should be confirmed to have local recurrence before any salvage treatment is considered. Attention should be given to the amount of time since primary treatment because some studies have shown that around 30% of positive biopsies at 12 months post RT had become negative biopsies at 24-30 months (1-4). Patients with rising PSA and biopsy-proven local recurrence after RT have organ-confined disease in only 20% to 39.5% of cases according to salvage RP literature (5, 6). Other challenges may be present in diagnosing local recurrence such as distinguishing findings of radiation changes from high-grade disease, which can lead to false positive results of the prostate biopsy.

Management of patients with local recurrence of prostate cancer after RT is challenging and there is no consensus on the best approach. According to the CaPSURE dataset, palliative treatment with androgen deprivation therapy (ADT) is the treatment more frequently used for biochemical recurrence following RT (2). Apart from systemic therapy, patients with biochemical recurrence after RT currently have the options of salvage radical prostatectomy (RP), salvage cryoablation (CA), salvage brachytherapy, and salvage high-intensity ultrasound (HIFU). The CaPSURE results show a high proportion of patients with locally advanced disease at the time that salvage therapy is considered. The rationale of salvage treatment is the potential to cure the disease instead of management through palliative treatment with androgen deprivation therapy and chemotherapy.

With the challenges of performing radical prostatectomy after RT such as fibrosis, tissue plane obliteration, high rates of urinary incontinence, major complications, rectal injuries, bladder neck contracture, and blood loss, urologists began offering ablative techniques for local recurrence of prostate cancer after RT.

Cryotherapy became an alternative for salvage RP in patients with locally recurrent prostate cancer following RT because of the potential to reduce morbidity (7). Third-generation cryotherapy devices reduce morbidity because they allow a more precise ice ball formation and the flexibility to place additional probes where needed to selectively target sites of recurrence (7-9). Also, the potential of focal ablation of the tumor instead of whole gland ablation is becoming a hot topic among ablative techniques (8, 9).

## PATIENT SELECTION FOR SALVAGE CRYOABLATION

Careful patient selection must be performed for patients with localized prostate cancer recurrence after RT and should include some patient and tumor characteristics before initial treatment. Careful patient selection has shown to have a better likelihood of a favorable outcome.

Ideally, patients should have a negative workup (negative bone scan and pelvic imaging) for metastatic disease and life expectancy >10 years.

Patients with pre-radiotherapy PSA <10ng/ml, Gleason score (GS) <8, clinical stage T1c or T2, and are low-risk showed to have a better outcome after salvage cryoablation (5).

Several studies demonstrated that pre-salvage cryoablation PSA >10ng/ml is a predictor of failure of salvage cryoablation and persistent PSA elevation despite initiation of ADT (5, 6, 10).

## ONCOLOGIC EFFICIENCY OF SALVAGE CRYOABLATION

Contemporary studies for salvage cryoablation show promising results and reduced morbidity compared to salvage RP. A large study from the COLD registry showed a 5-year biochemical disease-free survival (bDFS) rate of 58.9% using ASTRO criteria and 54.5% using Phoenix criteria (11). Cheetham et al. reported 10 years of follow-up for patients that underwent salvage cryoablation with biochemical recurrence of 52.2% using the Phoenix criteria (12).

Multiple studies consistently reported bDFS ranging from 34% to 74% with follow-up range of 1 to 10 years.

It is important to notice that there is no established definition of failure in the literature and bDFS rates vary based on the definition criteria of failure adopted by the study. Whole gland cryoablation of the prostate involves preservation of the urethra (with urethral warmer) where some of the prostatic tissue can remain, therefore undetectable PSA cannot be achieved. However, low PSA levels are acceptable, especially if they remain stable over time, and do not automatically reflect treatment failure.

### Complications of Salvage Cryoablation

The third generation of cryoablation devices significantly reduced the morbidity associated with the procedure compared to earlier generation devices. Cryoablation still has the same stigma associated with older devices, which turns some urologic oncologists away from offering this treatment option to their patients (7-9). Incontinence rates after salvage cryoablation decreased from 73% with older generation cryoablation device to 9.7% (5). Another study compared complications among different generation cryoablation devices, reporting a significant decrease in complications rates with the new technology (5). Another factor to consider is the correct identification of the external sphincter complex. The identification of the external sphincter complex by transrectal ultrasound is well established and cryosurgeons need to carefully identify and place the thermocouples correctly to prevent damage to the sphincter and avoid incontinence (13).

Acute and long-term complication rates following salvage cryoablation seem to be acceptable for the treatment of such a challenging patient population. Contemporary studies report mild to moderate urinary incontinence ranges from 6-13% and severe urinary incontinence from 2-4% for salvage cryoablation. Surgeons with more experience in cryosurgery can have much lower urinary incontinence rates (8-9%) and total incontinence in less than 1% of patients (5).

Postoperative urinary retention ranges from 2 to 21% of patients. It is very important to assess patient symptoms, urinary flow, and post-void residual before salvage cryoablation in order to proceed with workup for post radiation urethral stricture. The diagnosis of urethral stricture during the workup of recurrent prostate cancer can preclude salvage cryoablation due to risk of postoperative urinary retention and poor urinary function after treatment.

Erectile dysfunction (ED) is a known risk for patients that are eligible for salvage cryoablation; however, most patients considering salvage therapy already present significant ED following radiation therapy. ED following salvage total gland cryoablation ranges from 69% to 86% (5). Patients should be counseled that ED is a very likely complication for those who elect to proceed with salvage treatment. Salvage focal cryoablation is being considered for highly selected patients with local recurrence and promising results are been reported in a small series of patients (5).

One of the most feared complications of salvage cryoablation following radiation therapy recurrence is rectourethral fistula. The previous irradiated tissue, proximity to the rectum, and a more difficult delimitation of the prostate on live intraoperative transrectal ultrasonography are the challenges that surgeons will face when performing salvage procedures. The lack of experience with prostate ultrasonography and low-resolution equipment may account for the incorrect placement of the probes that can result in rectourethral fistulas. The incidence of rectourethral fistulas for salvage cryoablation is low (0-3%) and typically occurs within the first 25 patients treated by the cryosurgeon (5).

## PREDICTORS OF FAILURE OF SALVAGE CRYOBLATION

Spiess et al. identified pre-radiotherapy PSA and initial Gleason score as predictors of salvage cryoablation failure (10). Serum PSA (OR: 3.8) and biopsy Gleason score ≥8 at the time of diagnosis (OR: 2.9) were found to be strong predictors of biochemical recurrence.

Nadir PSA ≥0.6 ng/ml after salvage cryoablation was shown to increase the risk of developing biochemical recurrence at 12 months, and patients with Nadir PSA ≤0.6ng/ ml presented bDFS at 12, 24, and 36 months in 80%, 73.6%, and 67%, respectively. Levy et al. also showed that tumor burden measured by number of positive cores to prostate volume was also a prognostic factor for biochemical recurrence (14).

Data of pathology reports from salvage radical prostatectomy showed that viable tumor was found to be in the periurethral tissue in 67% of cases, 7% of which were located in direct contact with the urethra. An additional 17.4% of tumors were found within 2 mm of the urethral wall. These are the areas that cryosurgeons avoid ablating by locating the probe of the urethra at least 5 mm from the urethra and using the urethral warmer to prevent thermal urethral injury (15).

## FOCAL SALVAGE CRYOABLATION

Whole gland salvage cryoablation has been performed with acceptable outcomes. In an attempt to reduce even more treatment complications, some authors are proposing a technical modification to ensure tissue preservation by performing focal cryoablation of the area containing the tumor.

In order to accurately identify the area within the prostate containing the tumor recurrence, multiple image modalities have been used such as transrectal ultrasonography with target lesion biopsy and magnetic resonance imaging (MRI) with multiparametric approach (T2-weighted, diffusion-weighted, dynamic contrast-enhanced). The combination of image studies with transrectal or transperineal mapping biopsies (16-18).

Patients who are candidates for focal salvage prostate cryoablation need to have negative workup for metastatic disease, cancer recurrence restricted to one lobe of the prostate and small focus of recurrence. Most studies that evaluated feasibility of focal salvage cryoablation considered focal therapy as hemi-ablation of the gland, preserving the contralateral lobe and contralateral neurovascular bundle (16, 17).

The data regarding focal salvage cryo-ablation is still limited; however, results seem to be promising and comparable to whole gland ablation. bDFS rates range from 69%-100% in 1 year, 50%-72.4% in 3 years, and 46.5%-54.4% in 5 years (16).

## CONCLUSIONS

Overall, patients with local recurrence of prostate cancer following RT are associated with unfavorable prognosis. Advanced pathological stage is present at the time of diagnosis of cancer recurrence in two-thirds of patients.

More restricted criteria for biochemical recurrence are needed to improve early detection of patients with local recurrence. PSA monitoring alone may not be enough to detect cancer recurrence early following radiation therapy and the addition of image studies may benefit patients in an attempt to diagnose recurrence while the disease is still organ-confined. The combination of PSA, image studies, mapping biopsies, and possible new tumor markers may increase sensitivity and specificity of recurrence diagnosis in an early stage, allowing for a cure instead of subjecting patients to palliative treatment with ADT.

Currently, the majority of patients with cancer recurrence following RT receive palliative treatment with ADT despite multiple options for salvage treatment. Salvage radical prostatectomy is the most established procedure for salvage treatment; however, this procedure is technically difficult and associated with high morbidity.

Salvage cryoablation following RT recurrence is a promising alternative to salvage RP with lower morbidity to patients and similar mid-term oncological outcomes. Although long-term data is still pending, salvage cryo-ablation should be offered as a curative method with fewer complications compared to salvage RP to patients with confirmed local recurrence with no signs of metastatic disease.

Focal salvage cryoablation seems to be an alternative option for patients with unifocal cancer recurrence that are considering salvage therapy but want to minimize severe treatment complications as much as possible. Short-term data shows comparable bDFS of focal salvage cryoablation and whole gland salvage cryoablation. Data regarding salvage focal cryoablation is very limited and longer follow-up is required before focal salvage cryoablation can be considered.

Considering the benefits of ablative techniques (reduced treatment morbidity and comparable oncological outcomes compared to salvage RP), randomized clinical trials should be designed to determine treatment strategies for patients with cancer recurrence following radiation therapy.
